# Population Forecasts for Bangladesh, Using a Bayesian Methodology

**Published:** 2012-12

**Authors:** Md. Mahsin, Syed Shahadat Hossain

**Affiliations:** Institute of Statistical Research and Training; University of Dhaka, Dhaka 1000, Bangladesh

**Keywords:** Cohort component Method, Monte Carlo error, Gompertz model, Highest posterior density, Logistic model, Markov Chain Monte Carlo, Non-linear regression model, Population projection, WinBUGS

## Abstract

Population projection for many developing countries could be quite a challenging task for the demographers mostly due to lack of availability of enough reliable data. The objective of this paper is to present an overview of the existing methods for population forecasting and to propose an alternative based on the Bayesian statistics, combining the formality of inference. The analysis has been made using Markov Chain Monte Carlo (MCMC) technique for Bayesian methodology available with the software WinBUGS. Convergence diagnostic techniques available with the WinBUGS software have been applied to ensure the convergence of the chains necessary for the implementation of MCMC. The Bayesian approach allows for the use of observed data and expert judgements by means of appropriate priors, and a more realistic population forecasts, along with associated uncertainty, has been possible.

## INTRODUCTION

A widely-used method of forecasting the age and sex-specific population for future years, in which the initial population is stratified by age and sex and projections, is generated by application of survival ratios and birth rates, followed by an additive adjustment for net migration. To get this information, the behaviour of the related variables is analyzed based on the past data by statisticians, and then inferences are drawn from the analysis to make forecasts of the desired variable. At present, there exist two major paradigms in statistics, namely conventional (frequentist) and Bayesian statistics for the purpose of data analysis. Use of Bayesian methodology in the ﬁeld of data analysis is comparatively new and has found massive support in the last two decades from the experts belonging to various disciplines. Probably, the main reason behind the increasing support is its ﬂexibility and generality that allows it to deal with the complex situations. Besides, Bayesian method is typically preferred over classical approach in parameter estimation because of the intractable form of the likelihood function (1).

There are a number of methodologies used for population projections. One of the most popular methods is cohort component method which is based on the estimates about the future levels of fertility, mortality, sex composition, migration, and other parameters. Many studies have examined the relative performance of simple mathematical models, extrapolation based on time-series and cohort-component models of population forecasting. Most have found that constant growth mathematical models or standard time-series models of population growth are as least accurate as cohor component models (2-4).

The present study is not intended to assess the relative accuracy of various projection models. Rather, it only aims to investigate the usefulness of cohort component method in making the population projection for Bangladesh, using Bayesian approach. Bayesian analysis has been applied in cohort component model for providing a neat and transparent way of estimation. It provides probabilistic point estimates of the parameters, along with the highest posterior density interval (HPD) or Bayesian credible interval. Bayesian credible interval is a measure of uncertainty, and it is based on statistical theory and data on error distributions that provide an explicit estimate of the probability that a given range will contain the future population. This approach develops statistical prediction intervals to accompany population forecasts (5-7). Prediction intervals will provide extremely valuable information to data-users and will improve the quality of decision-making, based on population forecasts.

## LITERATURE REVIEW

A cohort component strategy of population projection is based on the logic of a general population-component methodology which examines separately the components of population change, fertility, mortality, and net migration. The cohort-component model of population projection (CCMPP) is perhaps the iconic method in demography (8-16). This classic method forwards, in time, a population defined by age according to a specified life table and set of age-specific fertility rates, taking into account the net migration at each age. A very basic equation can show the whole model:

P(t+n)=P(t)+Births−Deaths+Immigrants−Emigrants

where, *t* is the starting point of time; *n* is the projection interval; *P(t*) is the population-size at time *t*; and *P(t+n*) is the population size at time *t+n*. If we put immigrants and emigrants together, then we get:

*P(t+n)=P(t)+Births−Deaths+Net Migrants*

where, *Net Migrants=Immigrant−Emigrants*. A population grows through the addition of births and in-migrants and declines through the subtraction of deaths and out-migrants.

The term ‘fertility’ refers to the ability of an individual to give a livebirth (or births). This is equally applicable to a group or an entire population. Age-speciﬁc fertility rates are required to project the number of births in future fertility projections, which are made by projecting the course of TFR over time and translating this total fertility rate into age-speciﬁc fertility rates. In general, the projection of TFR is divided into assumptions regarding a level at which fertility eventually becomes constant in a country or a region and the path taken from current to eventual levels. Once fertility reaches its eventual level, the population will reach a stable age-structure and constant growth rate assuming that mortality and migration rates are also ﬁxed. If the eventual fertility level is at replacement level and net migration is zero, the growth rate will eventually be zero. Both projected pace of fertility decline and the assumed eventual fertility level are important for determining trends in population-size and age-structure. The lower the assumed eventual fertility level, the more important the pace of fertility decline becomes to projected population-size (17).

Births in cohort component models are typically projected by applying projected age-speciﬁc birth rates to projections of the female population by age. In this approach, the size and age composition of the female population of childbearing ages have a major impact on the projected number of births. Since most mothers for the ﬁrst 25 years of the projection period are already alive at the time the projection is made, the size and age composition of the female population are the most predictable elements in short-term fertility projections.

Time-series techniques have been used for projecting births or birth rates. Several authors have applied time-series methods by themselves, using autoregressive integrated moving average (ARIMA) methods to forecast total births (18-20). While these efforts yielded some insights into the use of time-series methods on fertility, the forecasts ignored the advantage of using cohort component methods (21). This omission was partially remedied by Lee (22-23) who applied time-series methods to TFR, the sum of all age-speciﬁc rates that occur in a given year. In our study, we have applied the Gompertz model, using Bayesian methodology to TFR.

The representation of mortality data via a parametric model has attracted the attention of actuaries, demographers, and statisticians for over a century. One of the most common models is that of logistic curve (13). In this paper, we adopt a Bayesian analysis to this curve, using MCMC technique to produce the posterior summaries required. For other Bayesian work relating to mortality smoothing and life-table construction (24-25), Carlin (26) used MCMC methods but not in a parametric curve modelling context.

## MATERIALS AND METHODS

Table 1 provides TFR in Bangladesh from 1991 to 2001, which have been used for a fertility model fit to making future fertility projections. Using these data and the Gompertz growth model, a WinBUGS program has been developed to make a Bayesian analysis of the data and to provide projections of the TFR of Bangladesh. In this paper, we follow the time-series tradition in developing a method to forecast TFR and then convert it to the age-speciﬁc fertility rates on the basis of base-year age-speciﬁc fertility rates. Multiplying these forecasts by forecasts of the size of the age-speciﬁc female population would then yield fertility forecasts derived from both time-series and demographic cohort component traditions. In this way, the advantages of the demographic tradition in taking account of the predictability of the size and age composition of the female population can be combined with the more statistically-rigorous time-series techniques of modelling the short-term variability of the age-speciﬁc fertility rates.

**Table 1.** Total fertility rate in Bangladesh from 1991 to 2001

Year (ti)	1991	1992	1993	1994	1995	1996	1997	1998	1999	2000	2001
TFR (Yi)	4.24	4.18	3.84	3.58	3.45	3.41	3.10	2.98	2.64	2.59	2.56

Source: Statistical Pocket Book; 2001-2007, Bangladesh Bureau of Statistics (BBS)

Let *Y*_*i*_ to denote TFR in Bangladesh in the year *t*_*i*_ (*i*=1, 2, …, 11) where *i* refers to successive censuses starting from 1991, for which *i*=1 and the data are given in Table 1. The most famous growth model is that of Gompertz (27) and is used for TFR where TFR *Y*_*i*_ in the year *t*_*i*_ has been assumed to follow normal distribution with respective means *h*_*i*_ and common precision τ. Non-informative priors have been assigned to all the parameters of the model. The nonlinear regression model for TFR is described as:

*Y_i_=h_i_+e_i_*

where *h_i_* is the deterministic part, and *e_i_* is the disturbance part; assuming the disturbance to be *e_i_~iid N* (0, τ), where τ is the precision (=1/variance), the fertility model and the non-informative priors might be defined as:

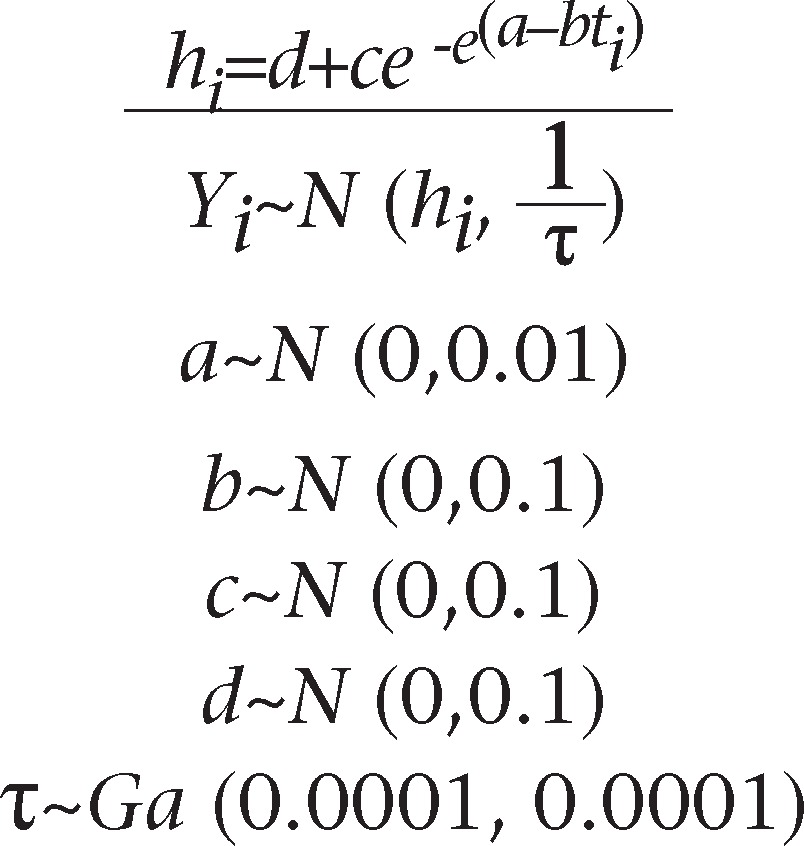


where *d* is the lower asymptote, *c* is the upper asymptote, *b* is the rate at which the fertility increases, and *a* is the parameter that determines the shape of the Gompertz curve. For Bayesian analysis, we need to provide prior distributions to all the parameters *a, b, c, d* and τ. A massive discussion on the choice of priors is also available in the BUGS manual (28).

Mortality projections are based on projecting future life-expectancy at birth for males and females, deﬁned as the average lifespan of a child born today if current age-speciﬁc mortality levels were held ﬁxed in the future. In developing countries where mortality remains high, future life-expectancy will be determined by the effciency of local health services, the spread of traditional (e.g. malaria) and new (e.g. AIDS) diseases, and the general standards of living and education. In this paper, we avoid the new epidemics (AIDS).

The life-expectancy at birth (average number of years lived by a newborn baby if he/she follows the current age-speciﬁc mortality patterns) is projected on the basis of the past experience of increase in the life-expectancy at birth. A logistic curve has been ﬁtted using trends in life-expectancy at birth, and it assumes that increase in life-expectancy at birth follows an S-shaped curve. The logic behind using logistic curve is that when the life-expectancy at birth is very low, the increase is expected to be slow due to poor health facilities. Once the health facilities are provided and with improvement in socioeconomic conditions, the life-expectancy increases at a faster rate. At the higher level of life-expectancy, the rate of increase is slow, and it would stabilize at the biological maximum. To project the population from one year to the next, survival rates by age and sex are needed and, to obtain future survival rates, future life tables may be constructed. Model life tables developed by United Nations (29), Coale and Demeny (30), Regional life tables, and South Asian model life tables, whichever is applicable for Bangladesh, should be used. In this study, South Asian model life table has been used.

Let *Q*_*ij*_ be the life-expectancy at birth for males and females of Bangladesh in the year *t*_*i*_ (*i*=1, 2, …, 21) where *i* represents time and *j* sex. The data were collected from office of the Bangladesh Bureau of Statistics [Sample Vital Registration System (SVRS): 2002, 2003, 2005-06, BBS], and a logistic growth model is used. In this model, the life-expectancy at birth *Q*_*ij*_ in the year *t*_*i*_ has been assumed to follow normal distribution with respective means *p*_*ij*_ and common precision τ_*j*_. Non-informative priors have been assigned to all the parameters of the model. The non-linear regression model for the population growth is described as:

*Q*_*ij*_=*p*_*ij*_+*ϵ*_*ij*_

where *p*_*ij*_ is the deterministic part, and *ϵ*_*i*_ is the random error part; assuming the error to be *ϵ*_*ij*_~*N* (0, τ_*j*_) where τ_*j*_ is precision, the mortality model and the non-informative priors are:


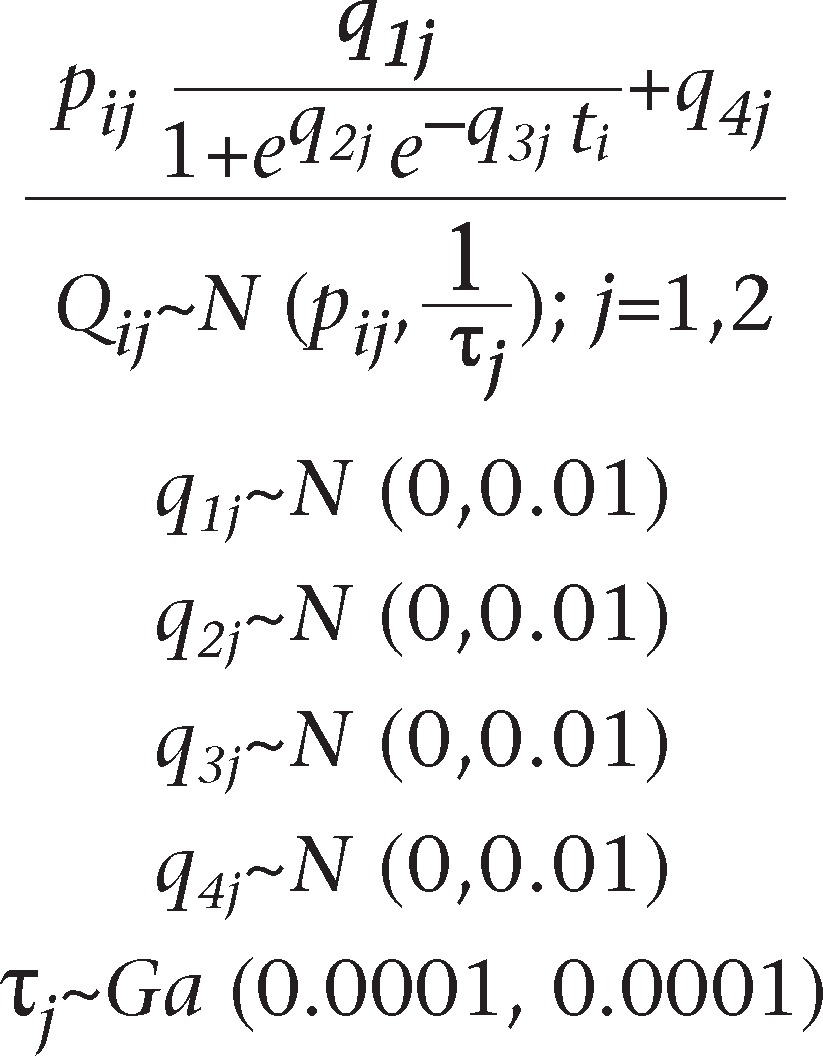


where *q*_*1*_ is the upper asymptote, *q*_*4*_ is the lower asymptote, *q*_*2*_ and *q*_*3*_ are the other parameters that define the shape of the logistic curve, and *e* is the base of the natural logarithm.

Future international migration is more difficult to project than fertility or mortality. Migration can be volatile since short-term changes in economic, social, or political factors often play an important role. In addition, projections are generally based on past trends and current policies since no single, compelling theory of migration exists; however, data on historical migration are sparse for Bangladesh. In this work, we assumed that the population is closed, i.e. no migration takes place, or even if it does, net effect is zero.

As for the sex ratio at births which divide the future number of newborns into male and female, the female to male ratio is set at 100:105 based on the results of the last ﬁve years, and it remains consistent from 2001 onward.

### Diagnostics

Bayesian approach faces serious computational difficulties due to likely involvement of complicated mathematical expressions in the posterior distributions. Many of these have been suitably addressed with greater ease, using MCMC methods. These methods enable us to carry out analysis on a wide range of Bayesian statistical models. More details with examples of the MCMC implementation in Bayesian inference can be found elsewhere (31-35). As an iterative tool, the MCMC methods are a class of algorithms for sampling from probability distributions based on constructing a Markov chain that has the desired distribution as its equilibrium distribution (33).

MCMC tool has been used in the WinBUGS to obtain the posterior distribution of the unknown parameters in the model. In the process, we need to run a number of chains for each parameter for a long time. When the chains have run sufficiently large number of iterations and have reached the stationary distribution, the samples obtained by further running of the chains are supposed to be drawn randomly from the posterior distribution of the parameter. WinBUGS provides a number of inbuilt diagnostics to assess the convergence of chains. For a more formal approach to convergence diagnosis, the software also provides an implementation of the techniques described in Brooks and Gelman (34), and a facility for outputting monitored samples in a format that is compatible with the CODA software (36).

In practice, WinBUGS allows multiple chains for each parameter to run simultaneously. Running multiple chains is a way to check the convergence of MCMC simulations. Two chains have been set in the model of this problem. When the diffierent chains do not provide sufficient mixing of chains even after a long run, it will be an evidence of lack of convergence of the chains. Once we are convinced that chains have been converged through the diagnostics, we will need to run the simulation for a further number of iterations to obtain samples that can be used for posterior inference. The more samples we save, the more accurate will be our posterior estimates. Once we have run enough updates and are satisﬁed with the history of the chains, we discard the earlier samples. We obtain the summary statistics only from the samples generated afterwards.

## RESULTS

The summary statistics of the estimated parameters of the fertility model after 10,000 initial updates were discarded and 80,000 updates were run after the initial burn-in is presented in Table 2. During these updates, none of the diagnostics indicated any symptom of non-convergence of the chains. The number of iterations required to run after the convergence of the chains is assessed on the basis of Monte Carlo error (MC error) for each parameter. MC error is an estimate of the difference between the mean of the sampled values (which we are using as our estimate of the posterior mean for each parameter) and the true posterior mean.

**Table 2.** Summary statistics of the node of fertility model

Node	Mean	SD	MC error	HPD region		
				2.50%	Median	97.50%
a	3.625	2.095	0.0831	-1.13	3.9	7.496
b	0.1878	0.07114	0.00247	0.08302	0.1769	0.3508
c	-3.488	1.23	0.04591	-6.537	-3.226	-1.857
d	5.058	0.7275	0.02837	4.168	4.871	6.952
σ	0.1011	0.03013	3.61E-04	0.06131	0.09513	0.1768

It has been suggested by the WinBUGS manual as a rule of thumb that the simulation should be run until the MC error for each parameter of interest is less than about 5% of the sample standard deviation, and this was followed in our analysis. From Table 2, it is obvious that MC errors for each parameter were less than 5% of the sample standard deviation.

**Figure 1.**
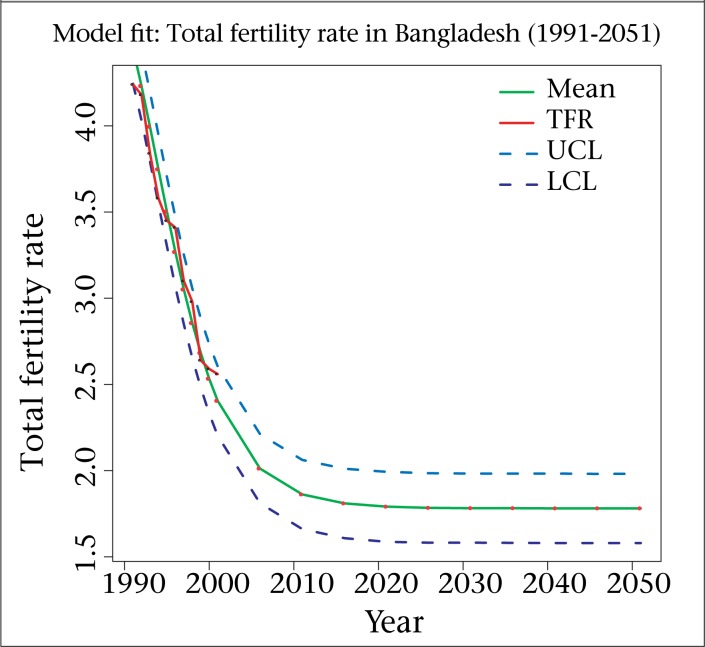
Fitted, projected and HPD region of the estimates on Gompertz model

Figure 1 illustrates a graphical presentation of the ﬁtting of the Gompertz model. The graph shows that the model provides a close ﬁt (the closeness of the smoothed line representing the estimated values and the dots showing the observed values) to the observed data. Dotted blue lines provide 95% HPD (highest posterior density) region.

Table 3 presents the summary statistics of estimated parameters of the mortality model on life-expectancy at birth for both males and females after discarding 10,000 initial updates and 70,000 updates were run after the initial burn-in. During these updates, none of the diagnostics indicated a symptom of non-convergence of the chains. While running our model with the WinBUGS, we have monitored ﬁve nodes *q_1_, q_2_, q_3_, q_4_,* and τ.

**Table 3.** Summary statistics of the node for life-expectancy at birth for both males and females

Node	Mean	SD	MC error	HPD region		
				0.025	Median	0.975
*q_1_*	Male	16.35	4.579	0.1705	9.589	15.65	27.05
	Female	18.21	4.442	0.1735	11.59	17.56	29.1
*q_2_*	Male	4.873	0.9117	0.03781	3.459	4.725	7.185
	Female	5.308	0.6038	0.02583	4.27	5.252	6.593
*q_3_*	Male	0.2561	0.06799	0.002848	0.1583	0.2424	0.4299
	Female	0.268	0.0471	0.00204	0.1887	0.2622	0.372
*q_4_*	Male	54.8	0.5047	0.01926	53.68	54.85	55.61
	Female	54.46	0.3151	0.01172	53.77	54.48	55.0
σ	Male	0.6012	0.1136	0.001698	0.4279	0.5849	0.8674
	Female	0.5063	0.09371	0.00126	0.3617	0.4934	0.7248

**Figure 2.**
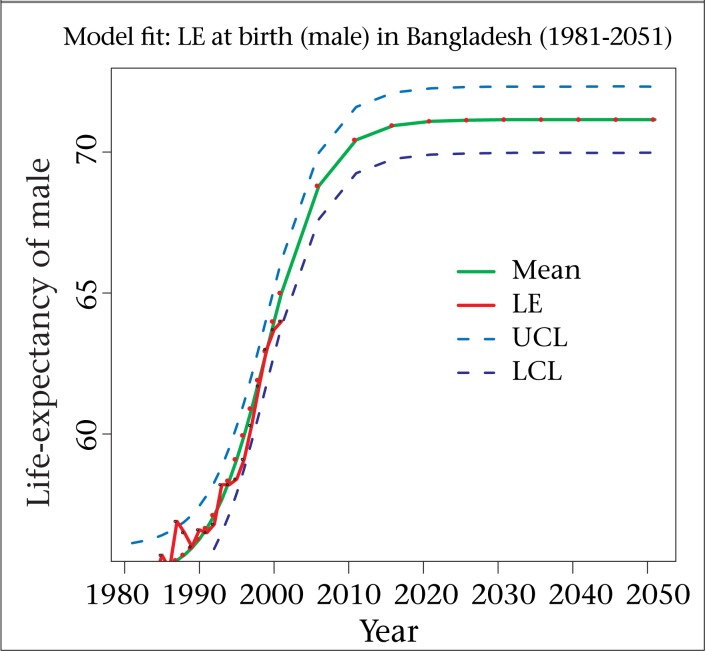
Fitted, projected and HPD region of the estimates under logistic model (male)

**Figure 3.**
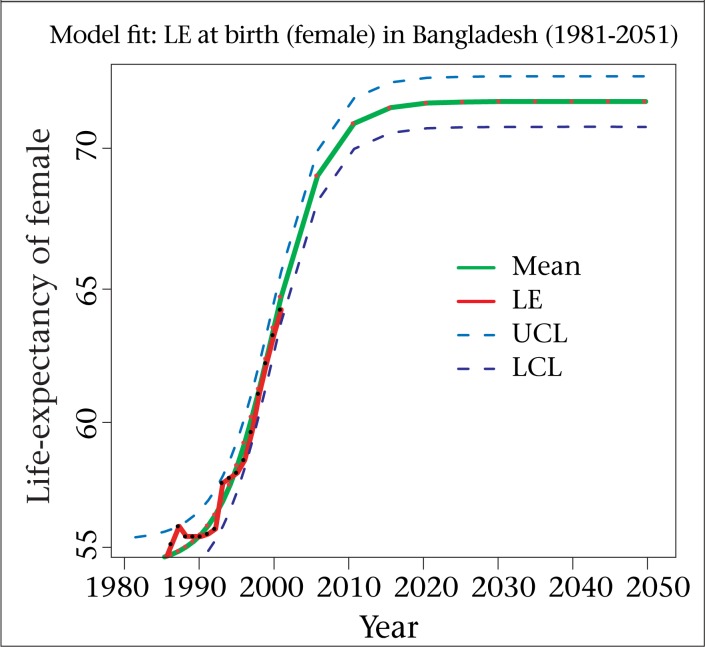
Fitted, projected and HPD region of the estimates under logistic model (Female)

The graphical presentation of the models fitted and forecasted to both males and females are depicted in Figure 2 and 3. The graphs show that the model provides a close ﬁt (the closeness of the smoothed line representing the estimated values and the dots showing the observed values) to the observed data. Dotted blue lines provide 95% HPD (highest posterior density) region. The graph of the projections approaches to S-shape, indicating the stabilization of the life-expectancy at birth for males as well as females.

## DISCUSSION

The ﬁnal calculations of cohort component method combine the results from the mortality, migration, and fertility modules. On the basis of the future forecasts of population growth components, the forecasted population of Bangladesh from 2006 to 2051 has been presented in Appendix.

The present study was an attempt to show the application and suitability of the MCMC tool in Bayesian data analysis for ﬁtting population data and making projection of the future population, using cohort component model. The use of Bayesian approach in fitting the components of growth models allows for further extensions over classical estimation methods, leading to a more realistic forecasts and associated uncertainty measures. The cohort component population projection method follows the process of demographic change and is viewed as a more reliable projection method than those that primarily rely on census data or information that reﬂect population change. In this paper, we had been presenting the basics of the implementation of the Bayesian data analysis with an illustration of the population projection. We have not performed the sensitivity analysis taking different prior distributions mainly because the selected priors were non-informative. These priors did not provide substantial information to the posterior distribution. However, they were necessary for the implementation of the Bayesian data analysis.

### Limitations

In this study, we are unable to provide future forecasts for the component of migration because of sparse data for Bangladesh. To overcome this problem, we have used a strong assumption, and this is the major drawback of our study. Apart from this shortcoming, the total fertility rate has declined to replacement level in 2010 and afterwards, which is unrealistic for Bangladesh but it is evident from Figure 2 and 3 that the mortality component has fitted very well. In both fertility and mortality models, we have applied non-informative priors, and it is also a limitation of this study. We hope to further explore these areas in future, using Bayesian methods motivated by the augments provided throughout this paper.

### Conclusions

Utilizing Bayesian methods to the growth components, a more realistic summary in population forecasts has been produced because it allows formal incorporation of expert judgement embodied in priors and, hence, alter the forecasted population characteristics and their levels of uncertainty. In this paper, we have applied non-informative priors to fertility and mortality models and, thus, a large level of uncertainty in the forecasted population is resulted. This level of uncertainty could be reduced through the inclusion of informative priors. Moreover, informative priors based purely on expert opinions regarding the future of population growth rates could have been included. Such prior information would result in further reductions in the estimated uncertainty due to added information in the parameter estimation and model-choice procedures.

**Appendix.** Age and sex-structure of the projected population (in thousands), 2006–2051

Age (years)	Sex	2001 (base)	2006	2011	2016	2021	2026	2031	2036	2041	2046	2051
All ages	Persons	123,851	133,436	143,515	154,212	164,899	174,494	182,384	188,577	193,432	196,933	198,964
	Males	63,895	68,699	73,700	78,957	84,267	88,942	92,739	95,687	97,989	99,671	100,682
	Females	59,956	64,737	69,815	75,255	80,632	85,552	89,645	92,890	95,443	97,262	98,282
0-5	Males	8,362	6,711	6,722	7,158	7,408	7,173	6,717	6,386	6,272	6,210	6,099
	Females	7,724	6,326	6,361	6,805	7,008	6,817	6,383	6,068	5,960	5,902	5,796
>5-10	Males	8,822	8,189	6,623	6,646	7,088	7,335	7,102	6,651	6,323	6,210	6,149
	Females	7,956	7,533	6,234	6,290	6,728	6,940	6,750	6,322	6,009	5,902	5,844
>10-15	Males	8,421	8,779	8,163	6,605	6,629	7,070	7,318	7,084	6,634	6,307	6,194
	Females	7,432	7,914	7,510	6,220	6,276	6,715	6,926	6,736	6,308	5,997	5,890
>15-20	Males	6,292	8,391	8,759	8,145	6,593	6,617	7,057	7,304	7,072	6,622	6,296
	Females	5,672	7,404	7,897	7,498	6,210	6,267	6,705	6,916	6,727	6,299	5,989
>20-25	Males	4,859	6,265	8,368	8,737	8,127	6,578	6,602	7,041	7,287	7,056	6,608
	Females	6,057	5,644	7,384	7,880	7,482	6,199	6,256	6,693	6,905	6,715	6,288
>25-30	Males	4,895	4,834	6,243	8,340	8,711	8,103	6,559	6,583	7,020	7,266	7,035
	Females	5,865	6,023	5,626	7,366	7,861	7,466	6,185	6,242	6,679	6,888	6,700
>30-35	Males	4,313	4,863	4,812	6,218	8,310	8,679	8,074	6,535	6,559	6,995	7,239
	Females	4,436	5,825	5,999	5,609	7,343	7,839	7,445	6,168	6,224	6,660	6,869
>35-40	Males	4,204	4,276	4,835	4,786	6,188	8,269	8,637	8,034	6,503	6,527	6,961
	Females	3,795	4,397	5,794	5,973	5,585	7,315	7,809	7,416	6,145	6,201	6,634
>40-45	Males	3,426	4,149	4,235	4,793	4,749	6,140	8,206	8,570	7,973	6,453	6,477
	Females	2,774	3,749	4,362	5,756	5,935	5,552	7,273	7,764	7,373	6,109	6,165
>45-50	Males	2,610	3,356	4,085	4,175	4,731	4,688	6,060	8,099	8,459	7,869	6,369
	Females	1,991	2,727	3,705	4,318	5,698	5,880	5,502	7,206	7,692	7,306	6,053
>50-55	Males	2,175	2,521	3,266	3,984	4,079	4,622	4,580	5,921	7,913	8,265	7,688
	Females	1,826	1,937	2,673	3,642	4,244	5,608	5,787	5,415	7,093	7,571	7,191
>55-60	Males	1,309	2,055	2,408	3,128	3,825	3,916	4,437	4,397	5,685	7,597	7,935
	Females	1,047	1,746	1,872	2,595	3,534	4,128	5,455	5,629	5,267	6,898	7,364
>60-65	Males	1,529	1,194	1,902	2,238	2,917	3,567	3,652	4,138	4,100	5,301	7,084
	Females	1,299	971	1,646	1,777	2,464	3,366	3,931	5,195	5,360	5,015	6,569
>65-70	Males	814	1,320	1,052	1,687	1,995	2,601	3,180	3,257	3,690	3,656	4,727
	Females	629	1,148	880	1,505	1,625	2,263	3,093	3,612	4,773	4,926	4,609
>70-75	Males	926	650	1,086	872	1,409	1,666	2,173	2,657	2,720	3,082	3,054
	Females	699	515	974	757	1,296	1,409	1,963	2,682	3,133	4,140	4,272
>75-80	Males	358	663	484	816	662	1,070	1,266	1,650	2,018	2,066	2,341
	Females	258	511	396	764	593	1,028	1,118	1,557	2,128	2,486	3,285
>80	Males	580	483	657	629	846	848	1,119	1,380	1,761	2,189	2,426
	Females	496	367	502	500	750	760	1,064	1,269	1,667	2,247	2,765
